# Minimally invasive beating heart technique for mitral valve surgery in patients with previous sternotomy and giant left ventricle

**DOI:** 10.1186/s13019-020-01171-6

**Published:** 2020-06-03

**Authors:** Hang Zhang, Hua-shan Xu, Bing Wen, Wen-zeng Zhao, Chao Liu

**Affiliations:** Department of Cardiovascular Surgery, The First Affiliated Hospital of Zhengzhou University, Zhengzhou, 450052 Henan China

**Keywords:** Minimally invasive, Beating heart technique, Redo mitral valve surgery, Previous sternotomy, Giant left ventricle

## Abstract

**Purpose:**

To analyze the efficacy of minimally invasive beating heart technique for mitral valve surgery in the cardiac patients with previous sternotomy and giant left ventricle.

**Methods:**

Eighty cardiac patients with previous sternotomy and giant left ventricle according to the diagnostic criteria that left ventricular end diastolic diameter (LVEDD) was ≥70 mm, who underwent mitral valve surgery at our center from January 2006 to January 2019 were analyzed. We divided all patients into minimally invasive beating heart technique group (*n* = 30) and conventional median resternotomy arrested heart technique group (*n* = 50) according to the surgical methods. Preoperative, intraoperative, and postoperative variables were compared between two groups.

**Results:**

Minimally invasive beating heart technique compared to the conventional median resternotomy arrested heart technique for mitral valve surgery in the cardiac patients with previous sternotomy and giant left ventricle had significant differences in operation time(*P* = 0.002), cardiopulmonary bypass (CPB) time(*P* < 0.001), intraoperative blood loss(*P* < 0.001), postoperative transfusion ratio(*P* = 0.01), postoperative transfusion amount(*P* < 0.001), postoperative drainage volume(*P* = 0.001), extubation time(*P* = 0.04), intensive care unit (ICU) stay time(*P* = 0.04) and postoperative hospital stay time(*P* < 0.001), but no significant differences in re-exploration for bleeding, postoperative 30-day mortality, postoperative complications and 6 months postoperative echocardiographic parameters.

**Conclusions:**

Using the method of minimally invasive beating heart technique for mitral valve surgery in the cardiac patients with previous sternotomy and giant left ventricle is effective and reliable, meanwhile reduce the operation time and CPB time, decrease the transfusion ratio and transfusion amount, shorten postoperative ICU stay and hospital stay time, promote the early extubation so that accelerate the patients’ early recovery.

All of these show a benefit of minimally invasive beating heart technique compared to conventional median resternotomy arrested heart technique.

## Background

With the increasing number of cardiac surgery, more and more cases of mitral valve reoperations are made because of various reasons.These reoperation patients tend to have a long course of disease, this could eventually lead to left ventricular enlargement and poor cardiac function. Especially for the patients with giant left ventricle according to the diagnostic criteria that left ventricular end diastolic diameter (LVEDD) is ≥70 mm, redo mitral valve surgery represents a clinical challenge due to a higher rate of perioperative morbidity and mortality. Formerly majority of cardiac surgeons preferred to choose original surgical approach through a median resternotomy for redo valve operations so that inevitably encountered risk of graft injuries, hemorrhage, the presence of dense adhesions, and complex valve exposure. Resternotomy may also be complicated in patients who had chest radiotherapy or previous sternal wound infections or in patients with vascular structures (ascending aorta, brachiocephalic vein, right ventricle and bypass vessels) that lie directly behind the sternum. Recently minimally invasive beating heart technique for redo cardic surgery had been safely performed successfully [1–7], which combined the availability and the advantage of a right-sided minithoracotomy approach without resternotomy and beating heart technique without aortic cross-clamping and cardiac arrest. The beating heart technique can interfere with the mechanisms of ischemia-reperfusion injury [8, 9] that may be advantageous particularly in patients with poor left ventricular functions [10–14]. The patients with giant left ventricle tend to merge poor heart function, we assume that such patients benefit from this operation. However, so far not seen minimally invasive beating heart technique for redo mitral valve surgery in patients with giant left ventricle at home and abroad for details. In the present study, we analyze primarily the efficacy of minimally invasive beating heart technique by comparing the preoperative, intraoperative, and postoperative variables of conventional median resternotomy arrested heart technique for mitral valve surgery in the cardiac patients with previous sternotomy and giant left ventricle.

## Materials and methods

### Patients

This study was approved by the Research Ethics Committee of the First Affiliated Hospital of Zhengzhou University. Eighty cardiac patients with previous sternotomy and giant left ventricle according to the diagnostic criteria that LVEDD was ≥70 mm, who underwent mitral valve surgery at our center from January 2006 to January 2019 were analyzed. If the patient had significant aortic regurgitation (the effective regurgitate orifice area greater than 1cm^2^) and (or) reoperation for immediate or early surgical failures (same hospital admission or less than 30 days) was excluded. From August 2011 to January 2019, 30 patients with giant left ventricle were reoperated through a minimally invasive approach in the right fourth intercostal space without aortic cross-clamping (minimally invasive beating heart group). From January 2006 to January 2019, 50 patients with giant left ventricle were reoperated through conventional median sternotomy with aortic cross-clamping and cardioplegic arrest (median resternotomy arrested heart group). Detailed preoperative demographics and patient characteristics are shown in Table 1.

**Table 1:** Preoperative demographics and patient characteristics

Features	Minimally invasive beating heart (*n* = 30)	Median resternotomy arrested heart (*n* = 50)	*P* Value
Demographics			
Age(years)	60.7 ± 6.8	60.2 ± 7.6	0.30
Male sex	18 (60.0%)	33 (66.0%)	0.59
Smoker	19 (63.3%)	34 (68.0%)	0.67
BSA(m^2^)	1.74 ± 0.08	1.74 ± 0.07	0.61
BMI(kg/ m^2^)	23.6 ± 0.9	24.0 ± 1.0	0.90
Comorbidities			
Congestive heart failure	6 (20.0%)	12 (24.0%)	0.68
Hypertension	13 (43.3%)	25 (50.0%)	0.56
COPD	2 (6.7%)	7 (14.0%)	0.52
Diabetes mellitus	2 (6.7%)	6 (12.0%)	0.70
Pulmonary hypertension	6 (20.0%)	15 (30.0%)	0.33
Atrial fibrillation	13 (43.3%)	21 (42.0%)	0.91
Cerebrovascular disease	1 (3.3%)	3 (6.0%)	1.0
Previous cardiac surgeries			0.60
MVP	8 (26.7%)	9 (18.0%)	
MVR	8 (26.7%)	18 (36.0%)	
MVR + TVP	4 (13.3%)	6 (12.0%)	
MVP + TVP	4 (13.3%)	3 (6.0%)	
MVR + AVR	4 (13.3%)	10 (20.0%)	
CABG	2 (6.7%)	4 (8.0%)	
NYHA functional class			0.64
I	4 (13.3%)	3 (6.0%)	
II	6 (20.0%)	15 (30.0%)	
III	14 (46.7%)	18 (36.0%)	
IV	6 (20.0%)	14 (28.0%)	
Logistic EuroSCORE	15.3% ± 5.4%	14.8% ± 5.4%	0.61
Predominant valve lesion			0.95
Perivalvular leakage	6 (20.0%)	9 (18.0%)	
Prosthesis dysfunction	2 (6.7%)	6 (12.0%)	
Rheumatic	4 (13.3%)	6 (12.0%)	
Endocarditis	6 (20.0%)	11 (22.0%)	
Perivalvular hyperplasia	8 (26.7%)	9 (18.0%)	
Bioprosthetic valve decay	4 (13.3%)	9 (18.0%)	
Echocardiography			
LVEF	0.43 ± 0.08	0.45 ± 0.09	0.27
LVEDD(mm)	75.8 ± 3.9	75.3 ± 3.6	0.52
LVESD(mm)	50.0 ± 5.3	49.2 ± 4.8	0.70
LVFS	0.31 ± 0.04	0.32 ± 0.04	0.81
Cardiothoracic Ratio	0.72 ± 0.05	0.71 ± 0.05	0.70
Moderate or greater TR	8 (26.7%)	14 (28.0%)	0.90

Continuous variables are expressed as means ± standard deviation

*Acronyms*: *BSA* Body surface area, *BMI* Body mass index, *COPD* Chronic obstructive pulmonary disease, *MVP* Mitral valvuloplasty, *MVR* Mitral valve replacement, *TVP* Tricuspid valvuloplasty, *AVR* Aortic valve replacement, *CABG* Coronary artery bypass grafting, *NYHA* New York Heart Association, *LVEDD* Left ventricular end diastolic diameter, *LVESD* Left ventricular end systolic diameter, *LVEF* Left ventricular ejection fraction, *LVFS* Left ventricular fraction shortening, *TR* Tricuspid regurgitation

### Minimally invasive beating heart technique

Under general anesthesia with a double lumen endotracheal intubation, patients were positioned with the right side of the chest slightly elevated. Transesophageal echocardiography (TEE) was performed and external defibrillation pads were placed in all patients. Sites of arterial and venous cannulation are described in Table 2. We preferred to use peripheral cannulation through the right internal jugular vein, right femoral vein, and right femoral artery after systemic heparinization, under TEE guidance. Especially the right internal jugular vein was percutaneously cannulated to better improve venous drainage for patients additionally undergoing tricuspid valve surgery. If only implemented the single femoral venous cannula that was inserted well into the superior vena cava (SVC) in order to avoid dislocation of the tip out of the SVC after left atrial retraction. Cardiopulmonary bypass (CPB) was established with vacuum assisted venous drainage with 30 mmHg negative pressure followed by a right anterolateral minithoracotomy through a 4 to 6 cm incision on the anterior axillary line via the right fourth intercostal space. The incision should be avoid as far as possible the breast for female patients. It had better to make a curved incision from the lower edge of the breast to protect the breast tissue. A rib spreader (Geister, Germany) was placed after a soft tissue retractor was inserted the incision. Then made two small incisions (1 cm in length) in the second and sixth intercostal space on the midaxillary line respectively. Carbon dioxide through the incision in the second intercostal space at 5 l per minute was continuously insufflated into the chest throughout the procedure to displace intracardiac air. A left ventricular drainage tube was placed through the incision in the sixth intercostal space. Under naked eye euthyphoria sheared longitudinally pericardium along anterior 2 cm of the right phrenic nerve, fixed anterior pericardium on the edge of the incision by silk thread and pulled posterior pericardium to posterolateral chest wall through two mininal incisions by suture. The aorta was not cannulated or clamped. At the beginning of experience, the patient’s body temperature was cooled to 27 or 28 °C to induce ventricular fibrillation. Subsequently, temperature was maintained between 32 and 33 °C to allow an operation on the empty beating heart [5, 7]. An aortic vent was always under continuous suction in the ascending aorta to evacuate air. With the heart perfused and beating, the left atrium was then immediately opened through interatrial groove or a trans-septal incision after a longitudinal right atriotomy in case of tricuspid surgery. For tricuspid surgery, dissociated the SVC and inferior vena cava (IVC) and completely blocked vena cava. In particular, we used 4–0 Prolene line with gasket to do the internal purse suture at SVC and IVC to right atrium opening that completely blocked vena cava, which avoided secondary injury of free vena cava because of fatal adhesion. An atrial retractor was placed through the fifth or sixth intercostal space parasternally and the left atriotomy was retracted anteriorly as a mass retraction. A left ventricular drainage tube was inserted right pulmonary vein that was used to maintain a clear operative field. The mitral valvuloplasty (MVP) or mitral valve replacement (MVR) was performed with interrupted pledgetted 2–0 braided polyester mattress sutures under direct vision. The tricuspid valvuloplasty (TVP) was performed by using the ring annuloplasty method. For coexisting paroxysmal or persistent atrial fibrillation, we preferred to use monopolar radiofrequency ablation maze approach that was easier in the minimally invasive setting. To prevent possible left atrial air embolism, the left atrium was filled with backflow of blood keeping the prosthetic or native valve open before closing the atriotomy. TEE was used to confirm proper valve and ventricular function and to ensure complete removal of air. The patient was disconnected from CPB after appropriate reperfusion and was decannulated. Placed pericardial drainage and chest drainage tubes, closed the femoral longitudinal incision and the thoracotomy in a standard fashion.

**Table 2:** Intraoperative patient characteristics

Features	Minimally invasive beating heart (*n* = 30)	Median resternotomy arrested heart (*n* = 50)	*P* Value
Operation time(hours)	3.75 ± 0.45	4.17 ± 0.62	**0.002**
Cross-clamp time(minutes)		86.3 ± 8.06	
CPB time(minutes)	130.2 ± 19.3	165.2 ± 27.6	**< 0.001**
Conversion to sternotomy	1 (3.3%)		
Re-exploration for bleeding	1 (3.3%)	4 (8.0%)	0.72
Intraoperative IABP	1 (3.3%)	4 (8.0%)	0.72
Blood loss(ml)	515 ± 188	697 ± 222	**< 0.001**
Temperature during CPB(°C)	32.1 ± 0.46	29.0 ± 1.47	**< 0.001**
Arterial cannulation			**< 0.001**
Ascending aorta	1 (3.3%)	44 (88.0%)	
Femoral artery	29 (96.7%)	6 (12.0%)	
Venous cannulation			
Bicaval	1 (3.3%)	44 (88.0%)	**< 0.001**
Femoral vein alone	6 (20.0%)	6 (12.0%)	
Jugulo-femoral	23 (76.7%)	0%	
Type of MV operation			0.93
MVR	26 (86.7%)	43 (86.0%)	
MVP	4 (13.3%)	7 (14.0%)	
TVP	8 (100%)	14 (100%)	1.0
Radiofrequency ablation	7 (53.8%)	15 (71.4%)	0.50

Continuous variables are expressed as means ± standard deviation. Bold values represent *P* values are considered to be statistically significant at <0.05

*Acronyms*: *CPB* Cardiopulmonary bypass, *IABP* Intra-aortic balloon pump, *MVP* Mitral valvuloplasty, *MVR* Mitral valve replacement, *TVP* Tricuspid valvuloplasty, *MV* Mitral valve

### Statistical analysis

Continuous variables are expressed as the mean ± standard deviation or median and the range of values (if non-normally distributed) and categoric variables are presented as proportions. Differences between groups were assessed by using the Pearson’s chi-square test or two-tailed *p*-value Fisher exact test where appropriate for categoric variables, the unpaired two-tailed t test for continuous variables (Mann-Whitney U test for non-normally distributed continuous variables). All statistical analyses were performed by using the IBM SPSS software package (version 20.0; SPSS, Chicago, IL, USA). A value of *p* < 0.05 was considered statistically significant.

## Results

### Demographics and preoperative and intraoperative characteristics

The patient demographics and preoperative characteristics in both groups were similar and not statistically significant differences (Table 1). The patients for redo mitral valve surgery tended to have older age, most of them were male and had a history of smoking. Previous cardiac surgeries were primarily mitral valve related diseases operations. The reoperation reasons were mainly mitral valve perivalvular leakage, mitral valve prosthesis dysfunction, rheumatic mitral valve disease, infective endocarditis, mitral valve perivalvular tissue hyperplasia, mitral bioprosthetic valve decay. The patients with giant left ventricle tended to have relatively low left ventricular ejection fraction (LVEF) (0.43 ± 0.08 vs 0.45 ± 0.09), New York Heart Association (NYHA) functional class above II accounted for the most part (66.7% vs 64.0%). Namely, these patients were more likely to have significant comorbidities at the time of surgery, for example, congestive heart failure, hypertension, chronic obstructive pulmonary disease (COPD), diabetes mellitus, pulmonary hypertension, atrial fibrillation, cerebrovascular disease and moderate or greater tricuspid regurgitation (TR), therefore mean preoperative logistic EuroSCORE predicted risk of operative mortality was quite high (15.3% ± 5.4% vs 14.8% ± 5.4%).

Intraoperatively, the operation times (3.75 ± 0.45 vs 4.17 ± 0.62 h, *P* = 0.002) and CPB times (130.2 ± 19.3 vs 165.2 ± 27.6 min, *P* < 0.001) were shorter in the minimally invasive beating heart group, blood loss (515 ± 188 vs 697 ± 222 ml, *P* < 0.001) was obviously decreased compared with median resternotomy arrested heart group (Table 2). There were 1 and 4 patients who required an intra-aortic balloon pump (IABP) to wean from extra-corporeal circulation in either group. One patient in the minimally invasive beating heart group underwent conversion to sternotomy because of extensive adhesions on the chest wall so that unable to single lung ventilation, at which time an aortic cross-clamp was placed. There were 1 and 4 patients who needed to re-exploration for bleeding in either group, but no statistically significance (3.3% vs 8.0%, *P* = 0.72). Arterial cannulation and myocardial protection strategies differed between the two groups. In the minimally invasive beating heart group, femoral arterial cannulation was almost used in 29 patients (96.7%), with the rest one undergoing ascending aortic cannulation (3.3%). On the contrary, in the median resternotomy arrested heart group, ascending aortic cannulation was used in 88.0% of patients (*n* = 44), and 6 patients (12%) underwent femoral arterial cannulation. Myocardial protection was achieved with ventricular fibrillation or superficial hypothermic empty beating heart (32.1 ± 0.46 °C core temperature) in beating heart surgery and performed with transthoracic aortic cross-clamp and cold crystalloid cardioplegia (29.0 ± 1.47 °C core temperature) in arrested heart. The distribution of valvular procedures performed and concomitant operative procedures is presented in Table 2. The most of patients for redo mitral valve surgery were performed MVR (86.7% vs 86.0%, *P* = 0.93). There were 8 and 14 patients who had concomitant moderate or greater TR in either group were all performed TVP by using the ring annuloplasty method. For coexisting paroxysmal or persistent atrial fibrillation, 7 patients were used monopolar radiofrequency ablation improved maze approach in the minimally invasive setting, however, 4 patients were used monopolar and 11 patients were used bipolar radiofrequency ablation improved maze approach in the conventional median resternotomy.

### Postoperative clinical outcomes

The early 30-day mortality was lower in the minimally invasive beating heart group, although not significant difference (6.7% [*n* = 2] vs 14.0% [*n* = 7], *P* = 0.52) (Table 3). Causes of death were multiple organ dysfunction syndrome (MODS)(*n* = 2) and coexist low cardiac output syndrome (LCOS)(*n* = 1) in the minimally invasive beating heart group. The postoperative complications in the minimally invasive beating heart group were all lower than that in the median resternotomy arrested heart group, such as stroke (0% vs 6.0%), acute renal failure (6.7% vs 14.0%), liver dysfunction (6.7% vs 10.0%), pulmonary complications (6.7% vs 12.0%), MODS (6.7% vs 14.0%), LCOS (3.3% vs 10.0%), ventricular fibrillation (6.7% vs 14.0%), cardiac tamponade (3.3% vs 6.0%), cardiac arrest (0% vs 4.0%), atrioventricular block (3.3% vs 8.0%), superficial wound infection (0% vs 4.0%), while there were no statistical significances. There was no statistical difference between the two groups in postoperative atrial fibrillation after radiofrequency ablation (36.7% vs 36.0%, *P* = 0.95). Fortunately, minimally invasive beating heart technique compared to the conventional median resternotomy arrested heart technique formitral valve surgery in the cardiac patients with previous sternotomy and giant left ventricle had significant differences in postoperative transfusion ratio (66.7% vs 90.0%, *P* = 0.01), median postoperative transfusion amount (2.0 vs 5.0 U, *P* < 0.001), median postoperative drainage volume (450 vs 800 ml, *P* = 0.001), median extubation time (13 vs 17 h, *P* = 0.04), median intensive care unit (ICU) stay time (16.5 vs 24.5 h, *P* = 0.04) and median postoperative hospital stay time (6 vs 9 days, *P* < 0.001). With patients’ agreement, we obtained the 6 months postoperative echocardiograms, which were available in all patients through consulting related cases data and telephone follow-up. The 6 months postoperative echocardiographic parameters (LVEDD, LVEF, cardiothoracic ratio, NYHA functional class) had a marked improvement compared with the preoperative circumstances, but there were no statistical significance between the two groups.

**Table 3:** Postoperative clinical outcomes

Features	Minimally invasive beating heart (*n* = 30)	Median resternotomy arrested heart (*n* = 50)	*P* Value
30-day mortality postoperative complications	2 (6.7%)	7 (14.0%)	0.52
Stroke	0%	3 (6.0%)	1.0
Acute renal failure	2 (6.7%)	7 (14.0%)	0.52
Liver dysfunction	2 (6.7%)	5 (10.0%)	0.92
Pulmonary complications	2 (6.7%)	6 (12.0%)	0.70
MODS	2 (6.7%)	7 (14.0%)	0.52
LCOS	1 (3.3%)	5 (10.0%)	0.51
Ventricular fibrillation	2 (6.7%)	7 (14.0%)	0.52
Cardiac tamponade	1 (3.3%)	3 (6.0%)	1.0
Cardiac arrest	0%	2 (4.0%)	0.53
Atrioventricular block	1 (3.3%)	4 (8.0%)	0.72
Superficial wound infection	0%	2 (4.0%)	0.53
Atrial fibrillation	11 (36.7%)	18 (36.0%)	0.95
Transfusion Ratio	20 (66.7%)	45 (90.0%)	**0.01**
Transfusion Amount(U)	2.0 (0 ~ 16)	5.0 (0 ~ 20)	**< 0.001**
Drainage Volume(ml)	450 (100 ~ 3000)	800 (250 ~ 4320)	**0.001**
Extubation time(hours)	13 (6 ~ 50)	17 (9 ~ 72)	**0.04**
ICU stay(hours)	16.5 (11 ~ 58)	24.5 (13 ~ 80)	**0.04**
Postoperative hospital stay(days)	6 (5 ~ 28)	9 (5 ~ 45)	**< 0.001**
6 months postoperative			
LVEDD(mm)	64.3 ± 3.3	64.4 ± 4.2	0.18
LVEF	0.55 ± 0.07	0.53 ± 0.08	0.50
Cardiothoracic Ratio	0.62 ± 0.04	0.63 ± 0.05	0.24
NYHA functional class			0.33
I	14 (46.7%)	18 (36.0%)	
II	13 (43.3%)	25 (50.0%)	
III	2 (6.7%)	4 (8.0%)	
IV	1 (3.3%)	3 (10.0%)	

Continuous variables are expressed as means ± standard deviation or medians with range. Bold values represent *P* values are considered to be statistically significant at <0.05

*Acronyms*: *LCOS* Low cardiac output syndrome, *MODS* Multiple organ dysfunction syndrome, *ICU* Intensive care unit, *LVEDD* Left ventricular end diastolic diameter, *LVEF* Left ventricular ejection fraction, *NYHA* New York Heart Association

## Discussion

Redo procedures usually involve sternal reentry, which has the potential hazardous injuries to the important structures and subsequent morbidity and mortality [15]. Furthermore, conventional cardiac arrest may predispose the dilated myocardium to ischemia-reperfusion injury and postoperative low cardiac output in the patients who have poor ventricular function and giant left ventricle for a long-standing valve diseases. Against the situation mentioned above, minimally invasive beating heart technique for mitral valve surgery in patients with previous sternotomy and giant left ventricle has emerged in this environment.

In our study, the intraoperative blood loss, postoperative transfusion ratio, postoperative transfusion amount and postoperative drainage volume were all reduced significantly in minimally invasive beating heart compared to that in median resternotomy. The greatest potential benefit of a right mini-thoracotomy is the avoidance of sternal re-entry and limited dissection of adhesions, avoiding the risk of injury to cardiac structures or patent grafts, and limiting the amount of postoperative bleeding [16]. With that in mind, it was not difficult to understand that the patients benefited reduced blood loss and less transfusions from minimally invasive beating heart. Moreover, the operation time and the CPB time in minimally invasive beating heart group were significantly shorter, whereas the shorter CPB time also reduced the need for perioperative blood transfusion, which was more important for redo patients. Of course, less CPB time decreased the release of inflammatory cytokines that contributed to a lower incidence of other comorbidities such as renal and liver insufficiency, pulmonary disease. Postoperative acute renal failure, liver dysfunction, MODS and pulmonary complications all declined in the minimally invasive beating heart, while there were no statistical significances. But there was statistical significance in the extubation time, the patients gained an early extubation which had a certain significance in preventing postoperative pulmonary infection and ventilator-induced lung injury. All the advantages we mentioned above significantly shortened postoperative ICU stay and hospital stay time so that accelerated the patients’ faster recovery. Romano and colleagues [6] concluded that redo right thoracotomy mitral valve surgery on the beating heart is associated with shorter bypass time, less transfusion requirements, shorter postoperative ventilation, and lower mortality. The mortality of patients with giant left ventricle was very high, the first cardic surgery death rate was 9% through median sternotomy in the study of D.HAN [17], the redo cardic surgery mortality would have been higher understandably. In our study, the logistic EuroSCORE predicted risk of operative mortality was high to 15.3% ± 5.4%. Fortunately, the postoperative 30-day mortality was 6.7% in minimally invasive beating heart group that was significantly lower than the expected mortality predicted by the logistic EuroSCORE, and it was less than 14% in median resternotomy arrested heart group, but there was no significant difference. Botta and colleagues [5] reported that two patients died in both groups (mortality was 4.5%) from multiorgan failure and CPB time was respectively 166 and 163 min, they asserted that there was no difference in biochemical or clinical outcomes from conventional surgery using aortic clamping and cardioplegic techniques. In our study, the 6 months postoperative echocardiographic parameters (LVEDD, LVEF, cardiothoracic ratio, NYHA functional class) had a marked improvement compared with the preoperative circumstances, but there were no statistical significances between the two groups. Murzi and colleagues [18] reported that 30-day mortality was 4.1% and reoperative mitral valve surgery could be safely performed through a right minithoracotomy with good early and late outcomes.

Currently, possible beating heart alternatives are performing the redo mitral valve operation with aortic endoballoon clamp [19] or an unclamped aorta [6, 12, 13] on the empty beating heart or ventricular fibrillation/fibrillating arrest while myocardial protection is achieved through continuous coronary perfusion. The big advantage of this continuous myocardial perfusion procedure is to decrease or eliminate myocardial damage caused by ischemia-reperfusion injury which follows standard manoeuvres of aortic cross clamping and clamp release [6, 8, 20], which may be advantageous particularly in patients with poor left ventricular functions [10–13, 21]. In the animal model, the morphology and function of the myocardial cells in ventricular fibrillation or sinus rhythm beating heart were all better than that in aortic occlusion during CPB [22]. In this study, we initially started to take the beating heart alternative with ventricular fibrillation, subsequently, we adopted beating heart technique with the empty beating heart that temperature was maintained between 32 and 33 °C. Some researchers believed that beating heart alternative with ventricular fibrillation approach was inferior to the empty beating heart owing to its reduction oxygen delivery to the subendocardium and the consequent suboptimal myocardial protection [6, 9, 21]. As normothermic perfusion was maintained, risk of coagulopathy was reduced and blood loss was usually much less than with hypothermic ventricular fibrillation [4, 6]. Therefore, this empty beating heart approach would be better helpful in patients with giant left ventricle tend to merge poor heart function. By keeping the heart beating, myocardial edema is decreased and function may be maintained, which may be of particular importance in these patients with already impaired ventricular function. These were good explanations for the postoperative morbidity of LCOS and ventricular fibrillation was lower in the minimally invasive beating heart group. As we all known, LCOS and ventricular fibrillation are leading causes of death in patients with giant left ventricle. This helps to further explain the lower postoperative 30-day mortality.

This beating heart method increased returned blood volume that influenced operation field, and increased cardiac attraction that contributed to corresponding blood damage augment [19]. It might be contraindicated if there was significant aortic insufficiency resulted in difficult to maintain a relatively bloodless operative field and sufficiently coronary perfusion. In our study, the patient had significant aortic regurgitation that the effective regurgitate orifice area greater than 1cm^2^ was excluded. In the event of concomitant mild aortic insufficiency, flows on CPB can be decreased and systemic temperature lowered in other studies [5]. Teruya and colleagues [4] used 2 drop-in suckers through the left atrial incision in this particular case, a left ventricular vent via the apex using mini-left thoracotomy was useful in preventing distention of the left ventricle. We obtained satisfactory results through a left ventricular drainage tube that was inserted right pulmonary vein. Meanwhile, we adopted remifentanil [23] and landiolol [24] that were helpful for heart rate reduction to prevent regurgitant blood flow from coming up to the operative field for very slight aortic insufficiency. Though we got a good view of surgery through the unremitting effects, it was difficult to perform MVP relative to MVR while the heart was kept perfused and beating. Therefore we had a very high probability (86.7%) of valve replacement that was similar to other studies [5]. On the other hand, the patients with large left ventricular heart valve disease in general had poor preoperative cardiac function and serious pathological lesion, so the surgery was mostly performed valve replacement or carried out valvuloplasty cautiously [17]. In the implementation of MVR, mitral posterior and subvalvular apparatus should be preserved as far as possible so that maximized to protect the left ventricular tension ring function and avoid the further expansion of the left ventricular transverse diameter [25].

It was noteworthy that the saline injection test was never been applied because it would pressurize the ventricle especially in valve repair. Another concern is the possibility of air embolism. An aortic vent was always under continuous suction in the ascending aorta and carbon dioxide was continuously insufflated into the chest to displace intracardiac air in this research. Additionally, the left atrium was filled with backflow of blood keeping the prosthetic or native valve open and the lungs were reinsufflated before closing the atriotomy to prevent possible left atrial air embolism. TEE was also used to ensure complete removal of air. We had achieved good results that there were no neurological complications caused by air embolism through using the methods mentioned above, which also had been confirmed in many other reports [7, 26]. In addition, minimally invasive beating heart approach can avoid systemic embolization caused by aortic clamping when severe aortic calcification. Some groups have reported increased stroke rates in patients undergoing the right minithoracotomy approach with retrograde arterial perfusion for redo mitral valve operation [27], but others hold the contrary opinion [28].

The limitations to the use of minimally invasive beating heart approach are mainly related to a prolonged learning curve that can increase the risk of patients at new centres and to the cost of the devices. At the beginning, there was one patient who needed to re-exploration for chest wall bleeding due to lack of experience. This kind of stupid mistake no longer occurred with the improvement of operation skill and experience. Although the operation of patient with giant left ventricle is difficult through minimally invasive thoracotomy, we should not ignore the great advantage of this method. As long as the lung function can be satisfied with one lung ventilation, we should try to take this kind of operation for the patients with giant left ventricle undergoing reoperation. In addition, there are several limitations that it is a small sample size and retrospective study at a single center in our study, long-term follow up data are also needed regarding the durability of this technique. Large-scale multi-center randomized controlled clinical trails are warranted to further validate the potential benefits and the limitations of this technique.

## Conclusions

In conclusion, minimally invasive beating heart technique has the potential to combine the benefits of minimally invasive access and continuous myocardial perfusion, which iseffective and reliable for mitral valve surgery in the cardiac patients with previous sternotomy and giant left ventricle. However, we could not demonstrate significant superiority in postoperative 30-day mortality and postoperative complications than could be achieved with a conventional median resternotomy and aortic cross-clamping and cardioplegic arrest. But the main advantages of this technique are that it avoid extensive surgical dissection, reduce the operation time and CPB time, decrease the transfusion ratio and transfusion amount, shorten postoperative ICU stay and hospital stay time, promote the early extubation so that accelerate the patients’ early recovery.

## Data Availability

The datasets used and/or analyzed during the current study are available from the corresponding author on reasonable request.

## Abbreviations

LVEDD: Left ventricular end diastolic diameter
CPB: Cardiopulmonary bypass
ICU: Intensive care unit
BSA: Body surface area
BMI: Body mass index
LVEF: Left ventricular ejection fraction
COPD: Chronic obstructive pulmonary disease
TR: Tricuspid regurgitation
MVP: Mitral valvuloplasty
MVR: Mitral valve replacement
TVP: Tricuspid valvuloplasty
AVR: Aortic valve replacement
CABG: Coronary artery bypass grafting
TEE: Transesophageal echocardiography
SVC: Superior vena cava
IVC: Inferior vena cava
NYHA: New York Heart Association
IABP: Intra-aortic balloon pump
MODS: Multiple organ dysfunction syndrome
LCOS: Low cardiac output syndrome
